# Identification of COL1A1 and COL1A2 as candidate prognostic factors in gastric cancer

**DOI:** 10.1186/s12957-016-1056-5

**Published:** 2016-11-29

**Authors:** Jun Li, Yuemin Ding, Aiqing Li

**Affiliations:** 1 Department of Gastroenterology, Sir Run Run Shaw Hospital, Zhejiang University, 3 East Qingchun Road, Hangzhou, Zhejiang China; 2 School of Medicine, Zhejiang University City College, 51 Huzhoujie, Hangzhou, Zhejiang China

**Keywords:** COL1A1, COL1A2, Gastric cancer, Premalignant, Prognosis

## Abstract

**Background:**

The role of type I collagen, composed of collagen type I alpha 1 (COL1A1) and collagen type I alpha 2 (COL1A2), has been studied in several cancers. However, the expression of COL1A1 and COL1A2 in malignant, premalignant, and normal gastric tissues and their clinical significances in gastric cancer have not been elucidated.

**Methods:**

Real-time quantitative PCR was performed in 55 malignant, 27 premalignant, and 19 normal tissues to measure COL1A1 and COL1A2 messenger RNA (mRNA) expression, and the correlations between COL1A1 and COL1A2 expression and clinicopathological parameters and patients’ survival rate were analyzed.

**Results:**

We found that COL1A1 mRNA expression was significantly upregulated in premalignant and malignant tissues than in normal tissues, whereas COL1A2 mRNA expression was significantly higher in malignant tissues than in premalignant and normal tissues. Moreover, COL1A1 expression was unrelated to clinicopathological parameters, while COL1A2 expression was positively related to tumor size and depth of invasion. Besides, higher COL1A1 and COL1A2 expression levels were related to lower overall survival.

**Conclusions:**

We find that COL1A1 might have its potential as a monitoring factor to screen early gastric cancer, and COL1A1 and COL1A2 might predict poor clinical outcomes in gastric cancer patients.

## Background

Gastric cancer ranks the third most common cause of cancer-related death and the fifth most common cancer in the world [1], while most gastric cancer patients are still diagnosed at the advanced stage, which means a relatively poor prognosis of overall survival [2]. Meanwhile, intestinal-type gastric adenocarcinoma, the most common form of gastric cancer, progresses through a cascade of gastric lesions known as Correa’s cascade [3], and the development from premalignant lesion to cancer is usually a gradual multi-step process. Thus, it is important to identify early biomarkers and/or prognostic factors for better outcomes.

Type I collagen, found in most connective tissue and embryonic tissue [4], is an important member of the collagen family which is a key structural component of the extracellular matrix. Typically, type I collagen is composed of two chains of collagen type I alpha 1 (COL1A1) and one chain of collagen type I alpha 2 (COL1A2) [5]. Recently, similar to the expressions of other members of the collagen family which is believed to be involved in carcinogenesis [6–8], abnormal expression of COL1A1 and COL1A2 has been reported in several cancers [9–11]. In addition, Zang et al. [12] indentified that COL1A1 and COL1A2 were differentially expressed in gastric cancer by complementary DNA (cDNA) microarray. However, the clinical significance of COL1A1 and COL1A2 in gastric cancer remains unclear, and the expression of COL1A1 and COL1A2 in normal epithelium, premalignant, and tumor lesions of the stomach is rarely mentioned.

The present study is to address COL1A1 and COL1A2 messenger RNA (mRNA) expression in malignant, premalignant, and normal tissues using real-time qualitative PCR. Then, analysis was performed to correlate COL1A1 and COL1A2 mRNA expression and clinicopathological parameters and overall survival time in gastric cancer.

## Methods

### Tissue specimens

Fifty-five malignant gastric tissues were collected during surgery (*n* = 45) and endoscopy examination (*n* = 10), while 27 premalignant gastric tissues and 19 normal gastric tissues were obtained during endoscopy examination in Sir Run Run Shaw Hospital (Hangzhou, Zhejiang, China). These gastric tissues were frozen immediately in liquid nitrogen and stored at −80 °C until RNA extraction. Written informed consent was obtained from all individual participants included in the study, and this research was approved by the Clinical Research Ethics Committee of Sir Run Run Shaw Hospital. The clinicopathological parameters included age, gender, tumor size, degree of differentiation, depth of invasion, and lymph node metastasis, and the follow-up period ranged from 1 to 60 months.

### RNA extraction and real-time quantitative PCR

Total RNA was extracted with an RNeasy Mini Kit (Qiagen, Hilden, Germany) according to the manufacturer’s instructions and was reversely transcribed into cDNA using PrimeScriptTM RT reagents kit (Takara, Japan). Real-time quantitative PCR reactions were performed using a SYBR® Premix Ex Taq™ kit (Takara, Japan) in a 7500 Real-time PCR System (Applied Biosystems, USA). The COL1A1-specific primers used were 5′-CTGCTGGACGTCCTGGTGAA-3′ (forward) and 5′-ACGCTGTCCAGCAATACCTTGAG-3′ (reverse), and the COL1A2-specific primers used were 5′-GAGGGCAACAGCAGGTTCACTTA-3′ (forward) and 5′-TCAGCACCACCGATGTCCAA-3′ (reverse). β-actin was served as an internal control, and the primers used were 5′-GTGGCCGAGGACTTTGATTG-3′ (forward) and 5′-AGTGGGGTGGCTTTTAGGATG-3′ (reverse). The expression of each gene was normalized to β-actin and was presented as a relative expression ratio (2^−ΔCt^, ΔCt = Ct_target_ − Ct_β-actin_).

### Statistical analysis

We assessed the differences of mRNA expression levels in the three groups and two groups by Kruskal–Wallis test and Mann–Whitney test, respectively. Correlation between mRNA expression and clinicopathological parameters was evaluated by Mann–Whitney test, while the Kaplan–Meier method was used to plot survival curves and the differences in survival rate were assessed by the log-rank test. A two-sided *p* value less than 0.05 was considered statistically significant. All data were analyzed using IBM SPSS Statistics 20.

## Results

### COL1A1 mRNA was significantly overexpressed in premalignant and malignant tissues, and COL1A2 mRNA expression was significantly higher in malignant tissues

**Fig. 1:**
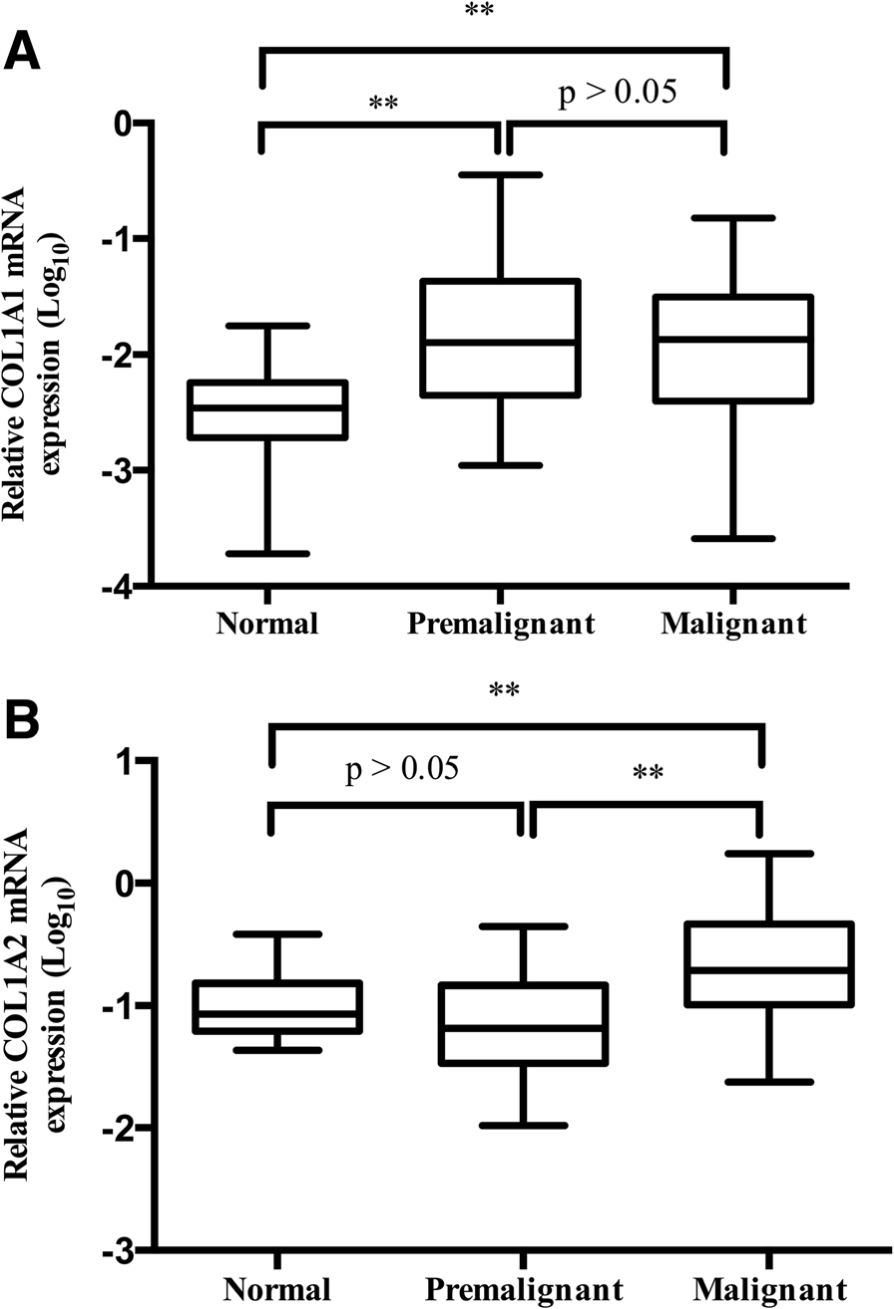
Expression of COL1A1 and COL1A2 in malignant, premalignant, and normal tissues of stomach. **a** Relative COL1A1 mRNA expression normalized to β-actin in malignant, premalignant, and normal tissues (**p < 0.01). **b** Relative COL1A2 mRNA expression normalized to β-actin in malignant, premalignant, and normal tissues (**p < 0.01)

Since there were significant differences of COL1A1 and COL1A2 expression among malignant, premalignant, and normal tissues, we further assessed the differences between two groups. As illustrated in Fig. 1a, COL1A1 mRNA was significantly increased in premalignant (*p* = 0.0007) and malignant tissues (*p* = 0.0019) compared to normal tissues, while no significant difference between premalignant and malignant tissues was observed (*p* = 0.7826). COL1A2 mRNA expression was significantly higher in malignant tissues than that in normal (*p* < 0.01) and premalignant tissues (*p* < 0.001), whereas there was no significant difference between premalignant and normal tissues (*p* = 0.1746) (Fig. 1b).

### COL1A2 mRNA expression was related to tumor depth of invasion and tumor size, and there was no relationship between COL1A1 mRNA expression and clinicopathological parameters

**Fig. 2:**
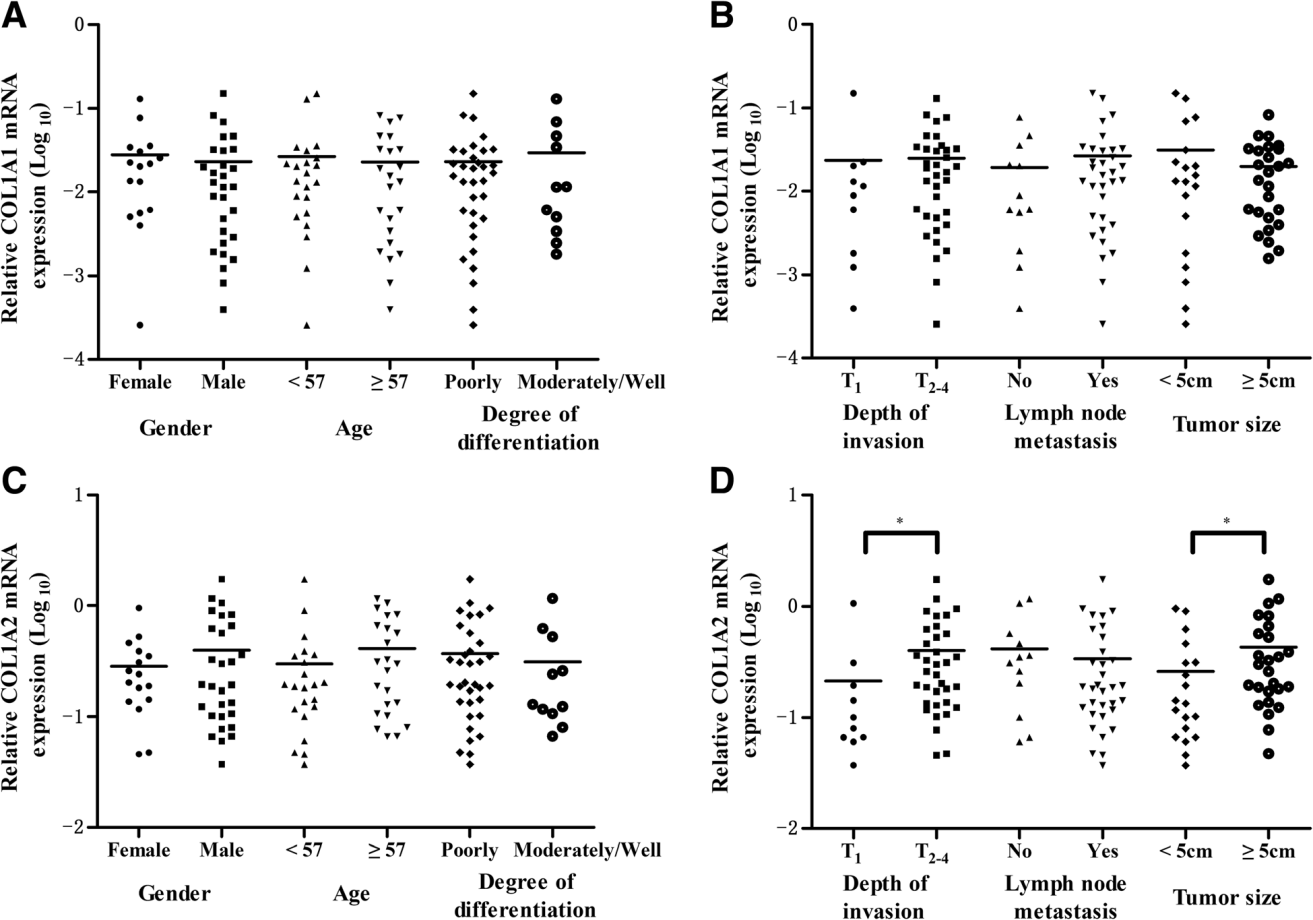
COL1A2 mRNA expression was related to tumor depth of invasion and tumor size. **a** COL1A1 mRNA expression was unrelated to gender, age, and degree of differentiation. **b** COL1A1 mRNA expression was unrelated to depth of invasion, lymph node metastasis, and tumor size. **c** COL1A2 mRNA expression was unrelated to gender, age, and degree of differentiation. **d** COL1A2 mRNA expression was significantly related to depth of invasion (*p < 0.05) and tumor size (*p < 0.05) but was unrelated to lymph node metastasis

The correlations between COL1A1 and COL1A2 expression and clinicopathological parameters were analyzed in 45 gastric cancer patients undergoing surgery. As shown in Fig. 2a, b, there was no significant correlation between COL1A1 mRNA expression and clinicopathological parameters regarding age, gender, depth of invasion, tumor size, degree of differentiation, and lymph node status (all *p* > 0.05), while no significant difference of COL1A2 expression was observed regarding gender, age, degree of differentiation, and lymph node metastasis (Fig. 2c, d), but COL1A2 expression was higher in the advanced stage and larger tumor size (*p* = 0.016 and *p* = 0.0385, respectively) (Fig. 2d).

### High COL1A1 and COL1A2 expression was related to poor overall survival rate

**Fig. 3:**
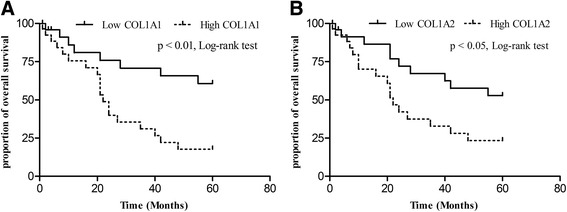
Kaplan–Meier analysis of overall survival for patients with gastric cancer (*n* = 55). **a** Patients with relatively high COL1A1 mRNA expression significantly correlated with poorer overall survival rate (*p* < 0.01). **b** Patients with relatively high COL1A2 mRNA expression have poorer overall survival rate than those with relatively low COL1A2 expression (*p* < 0.05)

We divided into two subgroups: low expression (*n* = 27) and high expression (*n* = 28). Univariate analysis by the Kaplan–Meier plot revealed that the overall survival time in patients with high COL1A1 expression (mean overall survival time 33.3 months; range 1–60 months) was significantly decreased compared to those with low COL1A1 expression (mean overall survival time 48.8 months; range 2–60 months) (*p* **=** 0.004) (Fig. 3a), while a similar result that patients with low COL1A2 expression (mean overall survival time 47 months; range 2–60 months) had a better overall survival rate compared to those with high COL1A2 expression was observed (mean overall survival time 35 months; range 1–60 months) (*p* = 0.028) (Fig. 3b).

## Discussion

Gastric cancer is one of the most aggressive cancers around the world, but the early diagnosis and overall survival time remain unfavorable [2]. Thus, identifying new prognostic factors and/or early tumor biomarkers is useful for better outcomes. In the present study, we found that COL1A1 and COL1A2 were overexpressed in gastric cancer and potent prognostic factors by showing their associations with an adverse prognosis in gastric cancer patients. In addition, COL1A1 might be a potential biomarker for early gastric cancer diagnosis.

Type I collagen, the major component of fibrillar collagen family, is believed to be involved in tumor invasion and progression [8, 13]. However, the expression levels of COL1A1 and COL1A2 in malignant tumors remain controversial. On one hand, frequent promoter methylation of COL1A1 was detected in renal cell carcinoma and hepatocellular carcinoma [9, 10], and COL1A2 was downregulated in melanoma [11], head and neck cancer [14], and bladder cancer [15]. On the other hand, COL1A1 and COL1A2 mRNA was upregulated in colorectal cancer and medulloblastoma [16, 17]. Consistent with previous reports [12, 18, 19], the present study demonstrated that COL1A1 and COL1A2 mRNA expression levels were highly expressed in gastric cancer specimens compared to normal gastric epithelium by real-time quantitative PCR analysis. In addition, gastric cancer is usually a multi-step gradual process, and COL1A1 mRNA expression was significantly higher than that in normal epithelium but did not significantly change from premalignant to tumor specimens. COL1A1 has also been reported to regulate proliferation and migration in BGC-823 gastric cancer cells [20]. Thus, these results indicated that COL1A1 may play a role in premalignant pathogenesis and may have its potential as a monitoring factor to screen early gastric cancer.

Although the prognosis of gastric cancer largely depends on the TNM stage at diagnosis [2], predicting patients with a poor prognosis and providing promising treatments are helpful for a better outcome. In the present study, we demonstrate the relationships between clinicopathological parameters and overall survival time and COL1A1 and COL1A2 mRNA expression in gastric cancer. No correlation was found between COL1A1 mRNA expression and the patients’ clinicopathological parameters, which is not consistent with the previous studies [21–23], while the overall survival rate was significantly lower in patients with high COL1A1 expression. The reason why COL1A1 expression did not correlate with depth of invasion and lymph node metastasis in this study needs to be further explored. COL1A2 mRNA expression was related to tumor size and depth of invasion, and high COL1A2 mRNA expression was positively associated with poor overall survival time. The present study suggests that COL1A1 and COL1A2 may be used as prognostic factors. Due to relatively smaller patients’ size, we failed to perform univariate and multivariate analysis of prognostic factors, and further studies with larger study sizes are needed to adequately confirm our result.

## Conclusions

We demonstrate that COL1A1 and COLA2 are overexpressed in gastric cancer, high COL1A1 might be a monitoring factor for early gastric cancer, and high COL1A1 and COL1A2 mRNA expression could be prognostic factors predicting overall survival time.

## Abbreviations

cDNA: Complementary DNA
COL1A1: Collagen type I alpha 1
COL1A2: Collagen type I alpha 2
